# TGF-β–Smad2/3 signaling in high-altitude pulmonary hypertension in rats: Role and mechanisms via macrophage M2 polarization

**DOI:** 10.1515/med-2025-1279

**Published:** 2025-10-09

**Authors:** Wende Ma, Yumei Ma, Yuting Bai, Cen Guo, Qibao Zhang, Xiaoling Su

**Affiliations:** Clinical Medical College, Qinghai University, Xining, 810007, Qinghai, China; Dongchuan Branch, Menyuan County Medical Community, Menyuan, 810399, Qinghai, China; Department of Digestive, Qinghai Provincial People’s Hospital, Xining, 810007, Qinghai, China; Department of Cardiology, Qinghai Provincial People’s Hospital, Xining, 810007, Qinghai, China

**Keywords:** high-altitude pulmonary hypertension, macrophages, transforming growth factor beta-β, interleukin-4, interleukin-10

## Abstract

**Objective:**

Transforming growth factor beta (TGF-β) is a key regulator of macrophage polarization, yet its role in high-altitude pulmonary hypertension (HAPH) remains poorly understood. This study aimed to explore the effects of TGF-β on macrophage polarization under high-altitude conditions and elucidate the molecular mechanisms driving macrophage phenotypic changes in HAPH, with potential therapeutic implications.

**Methods:**

A HAPH rat model was established by exposing rats to a hypoxic environment simulating 5,000 m altitude for 4 weeks. Rats were prophylactically treated weekly with the TGF-β inhibitor SB-431542. Pulmonary artery pressure and right ventricular hypertrophy index were measured to confirm model establishment. Hematoxylin and eosin staining, immunohistochemistry, and western blotting were used to assess TGF-β–Smad2/Smad3 signaling and macrophage polarization.

**Results:**

The HAPH group showed significantly increased pulmonary artery pressure and right ventricular hypertrophy index compared to controls. These changes were associated with elevated M2 macrophage levels, increased anti-inflammatory cytokines (IL-4 and IL-10), and enhanced TGF-β and Smad2/Smad3 signaling. TGF-β inhibition reversed these effects.

**Conclusion:**

The TGF-β–Smad2/Smad3 pathway promotes macrophage M2 polarization, driving HAPH progression through anti-inflammatory cytokine release. Inhibiting this pathway reduces M2 polarization and alleviates HAPH in rats, highlighting its therapeutic potential.

## 1 Introduction

In recent years, the number of individuals traveling to and residing in high-altitude areas has continued to rise, leading to an increased incidence of acute and chronic altitude sickness. High-altitude environments primarily induce acute and chronic mountain sickness through several pathways related to reduced oxygen levels in the body, increased vagus nerve tension, and abnormal immune system activation. High-altitude pulmonary hypertension (HAPH) occurs at altitudes over 2,500 m and is marked by pulmonary arterial remodeling, sustained increases in mean pulmonary arterial pressure (mPAP), chronic hypoxemia, and right ventricular hypertrophy, potentially resulting in fatal right heart failure. The primary pathophysiological mechanisms driving HAPH development involve maladaptive responses to the chronic low-pressure, hypoxic environments associated with high altitudes [1].

Research has shown that inflammatory responses are pivotal in the development of pulmonary hypertension (PH). Macrophages, which are key innate immune cells, regulate the levels of local cytokines and growth and chemotactic factors. Macrophages can polarize into pro-inflammatory M1 or anti-inflammatory M2 phenotypes through phenotypic switching, influencing the pathogenesis of various diseases [2,3]. M2 macrophages abundantly secrete transforming growth factor beta (TGF-β) and interleukin 10 (IL-10), which inhibit inflammation, promote tissue repair, regulate remodeling and angiogenesis, and maintain homeostasis [4]. In PH, the increase in anti-inflammatory cytokine levels can promote pulmonary arterial remodeling through various pathways such as those related to endothelial or smooth muscle cell proliferation and the secretion of macromolecules that comprise the extracellular matrix. Numerous studies have documented a notable rise in M2 macrophages in the lungs of rats with hypoxia- and lycorine-induced PH, as well as in patients with PH [5–7]. Nevertheless, the exact molecular mechanisms influencing macrophage polarization and potential phenotypic imbalance in HAPH are not yet fully understood. TGF-β is pivotal in various diseases due to its anti-inflammatory properties and its role in promoting abnormal vascular growth and repair. Evidence indicates that TGF-β can induce macrophage polarization toward the M2 phenotype. This study aimed to investigate the role and molecular mechanisms of the TGF-β–Smad2/Smad3 signaling pathway in mediating M2 macrophage polarization and its contribution to HAPH development in a rat model.

## 2 Materials and methods

### 2.1 Animals

This study received approval from the Animal Ethics Committee at Qinghai Province People’s Hospital, China (Approval No. (2023)-190).

Male Sprague-Dawley rats, weighing 200 ± 20 g, were provided by the Experimental Animal Center at Xi’an Jiaotong University’s Medical School, licensed under SCXK (Shaanxi) 2018-001. Animal experiments were performed at the Institute of Endemic Disease Prevention and Control located in Qinghai Province, China, and the molecular biology experiments were performed in the central laboratory of Qinghai Province People’s Hospital.

The rats were housed in a controlled environment with a 12 h light/dark cycle, a temperature maintained at 22 ± 2°C, and humidity kept between 40 and 60%. The rats were randomly assigned to three groups (*n* = 8 per group): a control group (maintained under normal conditions with weekly intraperitoneal saline injections of 1 mL), an HAPH group (exposed to a low-pressure hypoxic chamber simulating 5,000 m altitude for 4 weeks with weekly 1 mL saline injections), and an HAPH + SB-431542 group (subjected to the same hypoxic conditions with weekly intraperitoneal injections of 1 mL SB-431542 [5 mg/kg [8], dissolved in 10% DMSO, 40% PEG300, 5% Tween-80, and 45% saline], a selective TGF-β inhibitor).

### 2.2 Assessment of mPAP and determination of the right ventricular hypertrophy index

After 28 days of exposure to hypoxic conditions, the rats were put under anesthesia using sodium pentobarbital at a dose of 40 mg/kg. A polyethylene catheter was then inserted into the right ventricle and pulmonary artery through the right external jugular vein. The mPAP was recorded using a BL-420S Bio-signal Acquisition and Analysis System (Chengdu TME Technology Co, Ltd). The abdominal cavity was opened, and 3–5 mL of blood was carefully extracted from the abdominal aorta for subsequent analysis. Following blood collection, each rat was euthanized, and the thoracic cavity was opened to extract the heart and lung tissues. The left and right atrial tissues were removed to expose the ventricles, and the remaining heart was cut open along the inner wall of the septum. The RV and LV + S were isolated, dried with filter paper to eliminate excess moisture, and weighed using an electronic balance. The right ventricular hypertrophy index was calculated as the RV:LV + S weight ratio.

### 2.3 Hematoxylin and eosin (H&E) staining of lung arterioles

Lung tissue sections were fixed in 4% paraformaldehyde, dehydrated with graded ethanol, cleared in xylene, and embedded in paraffin for further analysis. The sections underwent deparaffinization, followed by hematoxylin staining for 10–20 min, eosin staining, dehydration, clearing, and mounting with neutral resin. Microscopic examination evaluated the lumen narrowing and wall thickness alterations in pulmonary arterioles. The study measured the wall thickness to lumen radius ratio of pulmonary arterioles and the wall cross-sectional area to total cross-sectional area ratio of the pulmonary vasculature.

### 2.4 Immunohistochemical analysis

Immunohistochemistry quantified the expression of CD68, CD86, CD206, inducible nitric oxide synthase (iNOS), and arginase 1 (Arg1) proteins in lung tissues. Briefly, paraffin-embedded lung tissue sections were deparaffinized, hydrated, subjected to antigen retrieval, and blocked to minimize non-specific labeling. Samples were incubated with primary antibodies (CD68: Proteintech, catalog number 28058-1-Ap, 1:1,000; CD86: Proteintech, catalog number 26903-1-Ap, 1:200; CD206: Proteintech, catalog number 18704-1-AP, 1:2,000; Arg1: Proteintech, catalog number 66129-1-IG, 1:3,000; iNOS: Proteintech, catalog number 80517-1-RR, 1:500) at room temperature for 1 h, followed by a PBS wash. Subsequently, secondary antibodies (Proteintech) were applied, and samples were incubated at room temperature for 20–30 min. Sections were subsequently washed with PBS, and 3,3′-diaminobenzidine was applied as a chromogen. The sections underwent hematoxylin counterstaining, alcohol dehydration, washing, drying, xylene clearing, neutral resin mounting, further drying, and were then photographed microscopically. ImageJ software was utilized for semi-quantitative analysis.

### 2.5 Western blotting

Western blotting was performed to assess the expression of signaling proteins TGF-β, Smad2, and Smad3; pro-inflammatory markers IL-6R and TNF-α; and anti-inflammatory cytokines IL-4 and IL-10 in lung tissue. Proteins (10 µg per lane) were separated using 10% SDS-PAGE and transferred to a PVDF membrane. The membrane was blocked at room temperature for 2 h using 5% non-fat milk, followed by washing with tris-buffered saline with Tween 20 (TBST). It was then incubated overnight at 4°C with primary antibodies (TGF-β: Proteintech, catalog number 21898-1-Ap, 1:3,000; Smad2: Proteintech, catalog number 12570-1-Ap, 1:6,000; Smad3: Abclonal, catalog number A19115, 1:10,000; IL-6R: Proteintech, catalog number 23457-1-Ap, 1:700; TNF-α: Proteintech, catalog number 17590-1-Ap,1: 1,000; IL-4: Proteintech, catalog number 66142-1-Ig, 1:1,000; IL-10: Proteintech, catalog number 60269-1-Ig, 1:5,000) washed with TBST, and incubated with secondary antibodies (Proteintech, catalog number RGAR001, 1:6,000) at room temperature for 1.5 h. Protein bands were visualized using an enhanced chemiluminescence system and captured with a gel imaging system. ImageJ software was utilized for image analysis.

### 2.6 Statistical analysis

The data were shown as the average ± standard deviation (SD). All statistical tests were conducted using SPSS software, version 25.0. Groups were compared using one-way ANOVA, followed by *post hoc* least significant difference tests. A *p*-value under 0.05 was considered statistically significant.

## 3 Results

### 3.1 Assessment of mPAP and determination of the right ventricular hypertrophy index

**Figure 1:**
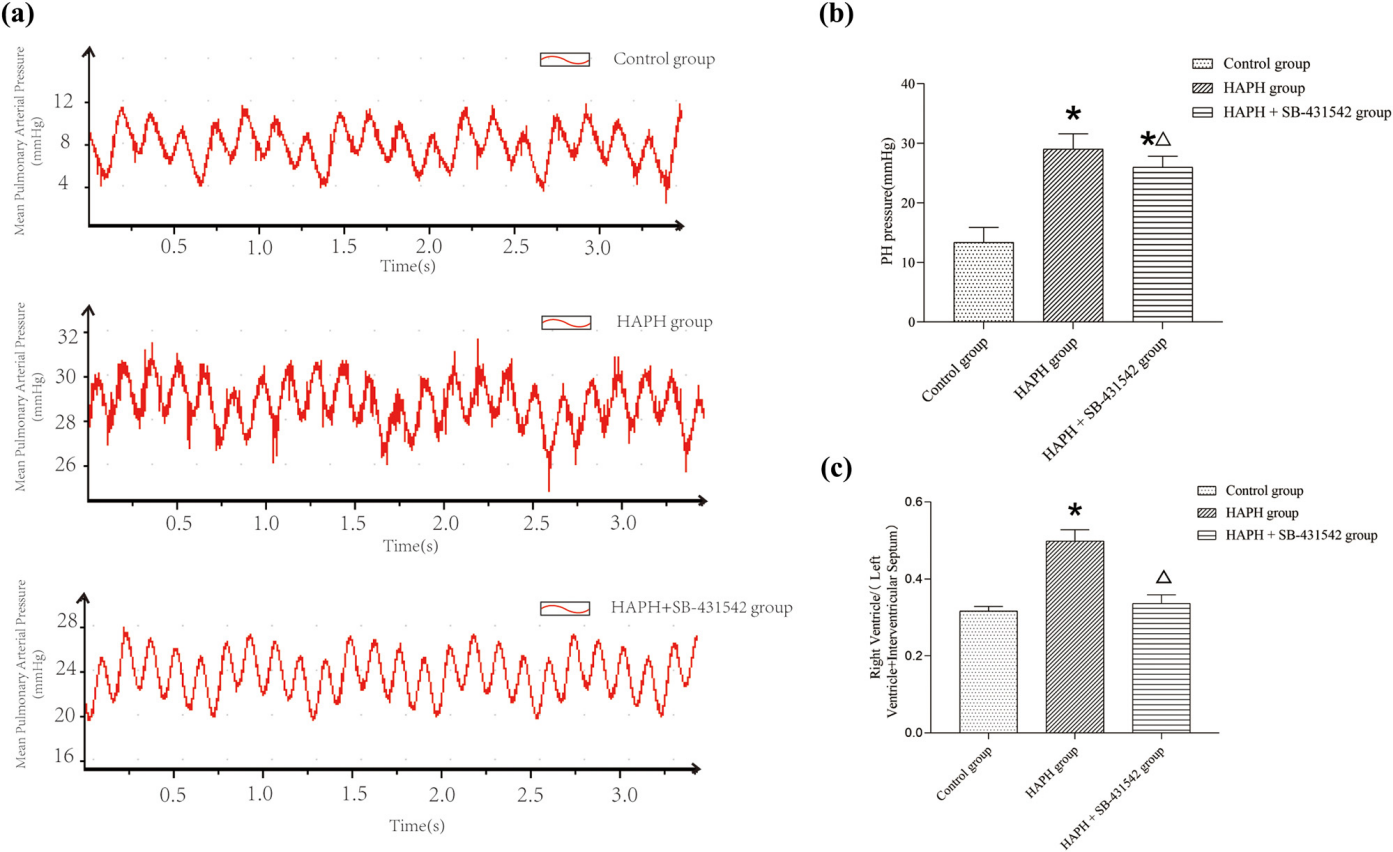
mPAP and right ventricular hypertrophy index in rats. Measurement of the mPAP via right heart catheterization (a). Mean pressure of the pulmonary artery (b). Assessment of the right ventricular hypertrophy index (c). Values are presented as mean ± standard deviation. *p < 0.05 compared to the control group; Δ*p* < 0.05 compared to the HAPH group.

The figure presents the results of right heart catheterization measurements of mPAP and right ventricular hypertrophy index values (Figure 1a–c). The HAPH group exhibited significantly elevated mPAP and right ventricular hypertrophy index compared to the control group; these increases were notably reduced in the HAPH + SB-431542 group following TGF-β inhibition.

### 3.2 Pathological changes in the small pulmonary arteries of HAPH model rats

**Figure 2:**
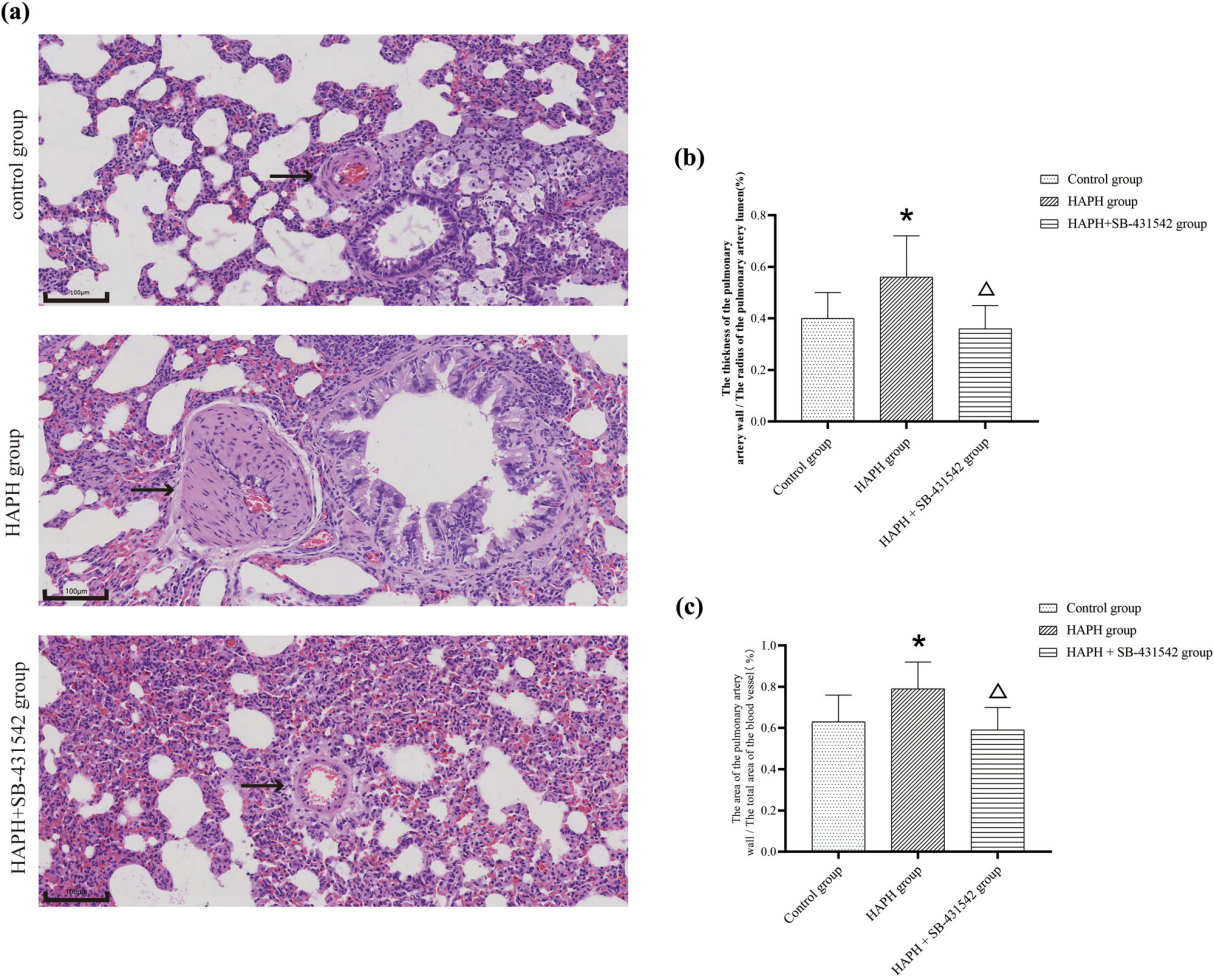
Pathological changes in the lung tissue of HAPH model rats. H&E staining for the detection of morphological changes. The black arrows indicate the pulmonary artery; scale bar, 100 μm (a). Ratio of the pulmonary artery wall thickness to the lumen radius (b). Ratio of pulmonary artery wall area to total pulmonary vessel area (c). Values are presented as mean ± standard deviation. *p < 0.05 compared to the control group; Δ*p* < 0.05 compared to the HAPH group. Abbreviations: HAPH, high-altitude pulmonary hypertension; H&E, hematoxylin and eosin; SD, standard deviation.

H&E staining was performed to assess and compare pathophysiological changes between groups (Figure 2a). The control group rats showed neither lumen narrowing nor wall thickening in the small pulmonary arteries. In contrast, the HAPH group rats demonstrated significant narrowing of the lumen and thickening of the arterial walls; these HAPH-induced changes were absent in the HAPH + SB-431542 group rats, who were treated with the TGF-β inhibitor. The HAPH group exhibited a significantly higher wall thickness to lumen radius ratio in small pulmonary arteries compared to the control group. In contrast, the HAPH + SB-431542 group’s ratio was not significantly different from the control group but was significantly lower than that of the HAPH group (Figure 2b). The HAPH group exhibited a significantly higher ratio of the small pulmonary artery wall cross-sectional area to the total pulmonary vasculature area compared to the control group. In contrast, the HAPH + SB-431542 group’s ratio was similar to the control group and significantly lower than the HAPH group (Figure 2c). The study suggests that high-altitude-like low-pressure and hypoxic conditions lead to narrowing and thickening of small pulmonary arteries in rats, effects that can be mitigated by the TGF-β inhibitor SB-431542.

### 3.3 Expression levels of the macrophage phenotypic markers CD68, CD86, CD206, iNOS, and Arg1 in lung tissue

**Figure 3:**
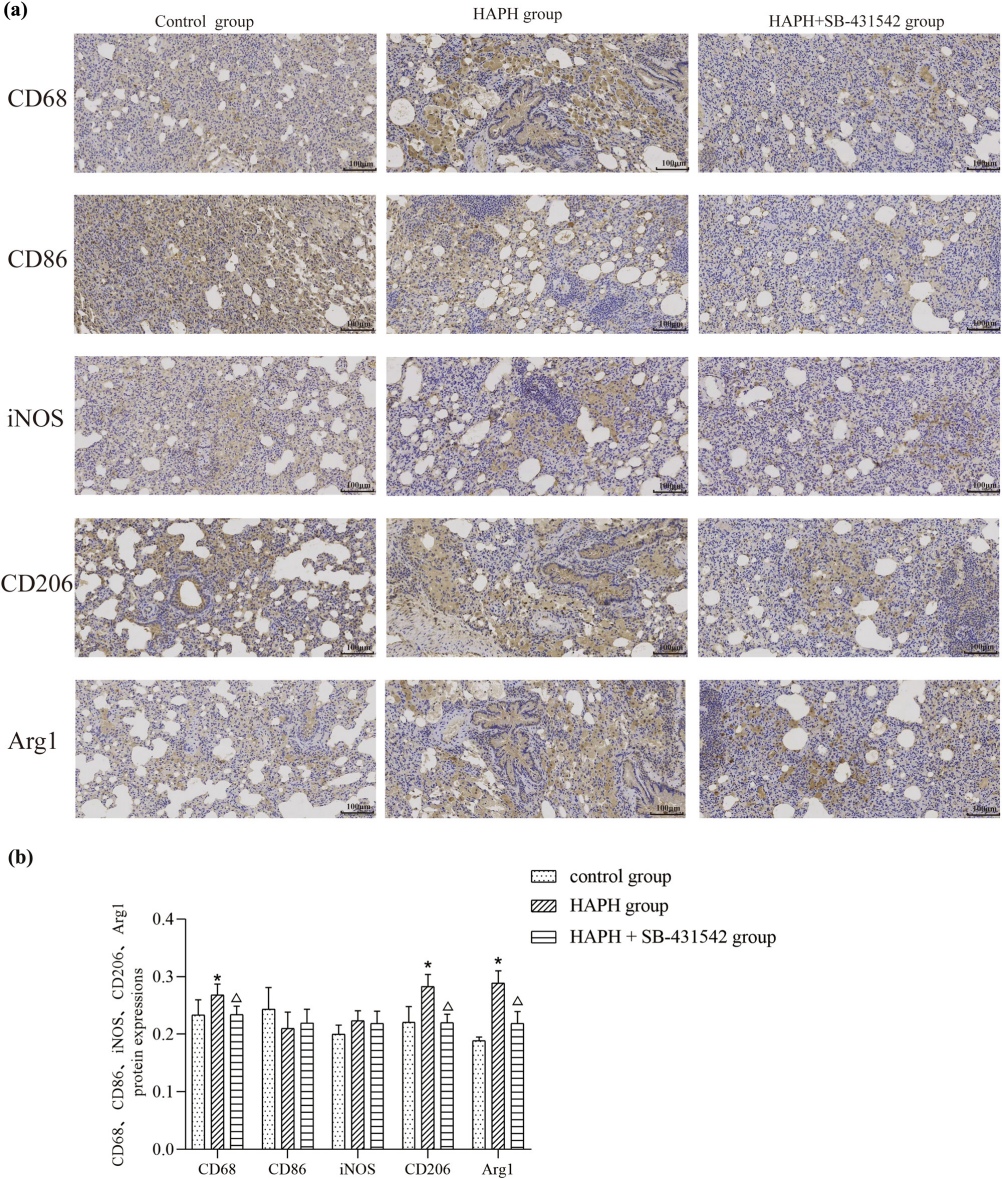
Representative images showing CD68, CD86, CD206, iNOS, and Arg1 expression; scale bar, 100 μm (a). Quantitative analysis of positive cell density (b). Values are presented as mean ± standard deviation. *p < 0.05 compared to the control group; Δ*p* < 0.05 compared to the HAPH group. Abbreviations: CD, cluster of differentiation; iNOS, inducible nitric oxide synthase; Arg1, arginase 1; SD, standard deviation.

Immunohistochemical labeling of phenotypic markers was performed to assess changes in macrophage polarization (Figure 3a and b). CD68 expression, indicative of macrophage presence, was notably increased in the HAPH group relative to the control group, but remained unchanged in the HAPH + SB-431542 group. The HAPH group exhibited a significantly higher number of M2 macrophages (CD206 + Arg1 + cells) compared to the control group, while the HAPH + SB-431542 group showed no difference. The M1 macrophage count (indicated by CD86 + iNOS + cells) in both the HAPH and HAPH + SB-431542 groups showed no significant difference compared to the control group. The HAPH + SB-431542 group exhibited significantly fewer overall macrophages (CD68+) and M2 macrophages (CD206+ Arg1+) compared to the HAPH group, while the number of M1 macrophages (CD86+ iNOS+) showed no significant difference between the groups. These findings indicated that exposure to low-pressure and hypoxic conditions, mimicking those at high altitudes, can lead to an imbalance in macrophage polarization, with a predominance toward the M2 phenotype, and this phenotypic shift can be attenuated by administration of the TGF-β inhibitor SB-431542.

### 3.4 Expression levels of TGF-β, Smad2, and Smad3 in pulmonary tissue

**Figure 4:**
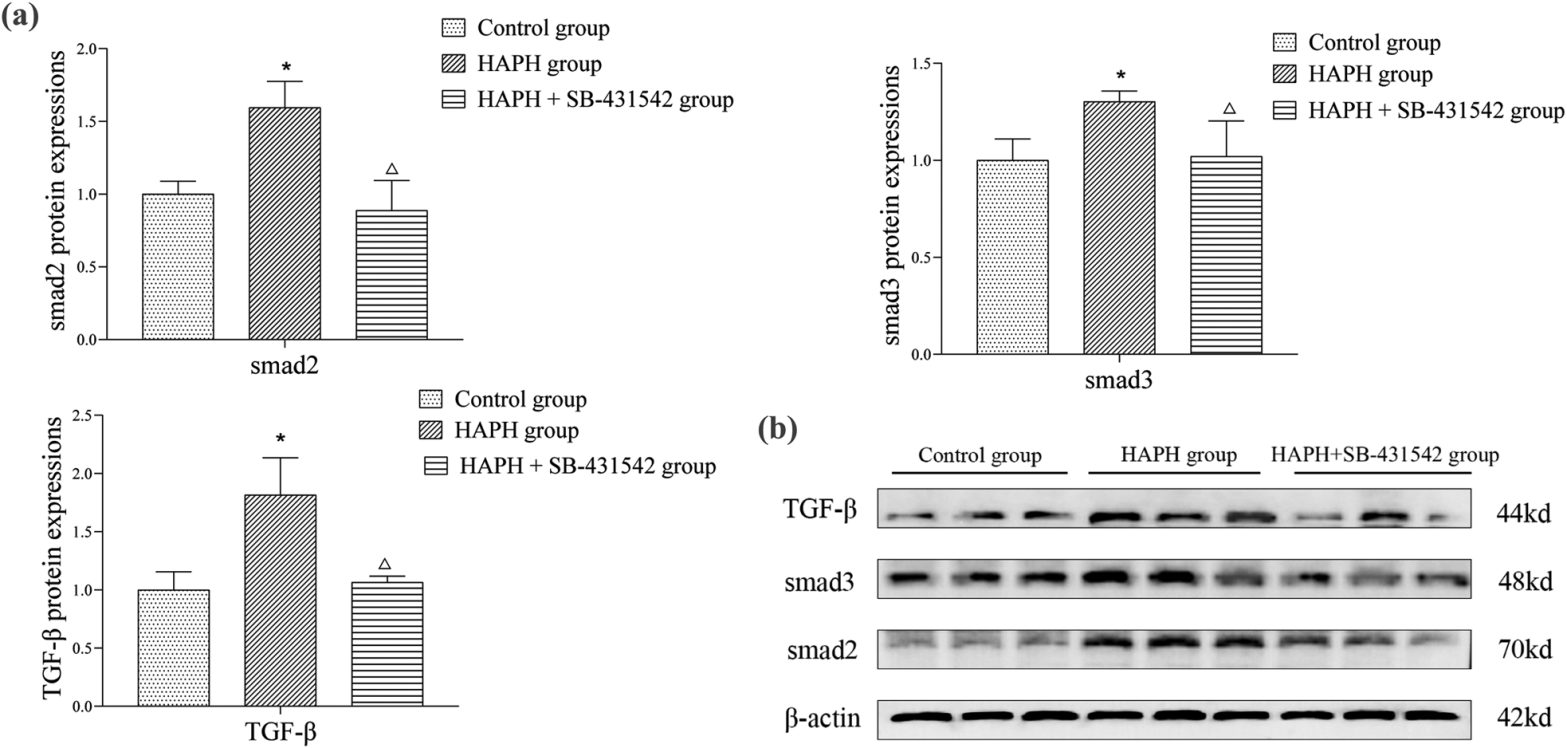
Quantitative analysis of TGF-β, Smad2, and Smad3 expression levels in lung tissue (a). Representative western blot bands of target proteins (b). Measurement of protein expression levels. Values are presented as mean ± standard deviation. *p < 0.05 compared to the control group; Δ*p* < 0.05 compared to the HAPH group. Abbreviations: TGF-β, transforming growth factor beta; SD, standard deviation; and HAPH, high-altitude pulmonary hypertension.

The western blot analysis of TGF-β, Smad2, and Smad3 expression levels in lung tissues is shown in Figure 4a and b. The HAPH group exhibited significantly elevated levels of TGF-β, Smad2, and Smad3 compared to the control group, whereas the HAPH + SB-431542 group showed no significant differences. The HAPH + SB-431542 group exhibited a significant downregulation of TGF-β, Smad2, and Smad3 expression compared to the HAPH group. These findings indicated that the TGF-β, Smad2, and Smad3 signaling pathways are involved in the development of PH induced by exposure to low-pressure and hypoxic conditions, mimicking those at high altitudes in rats, and these changes could be attenuated by administration of the TGF-β inhibitor SB-431542.

### 3.5 Expression levels of inflammatory markers IL-6R, TNF-α, IL-4, and IL-10 in lung tissue

**Figure 5:**
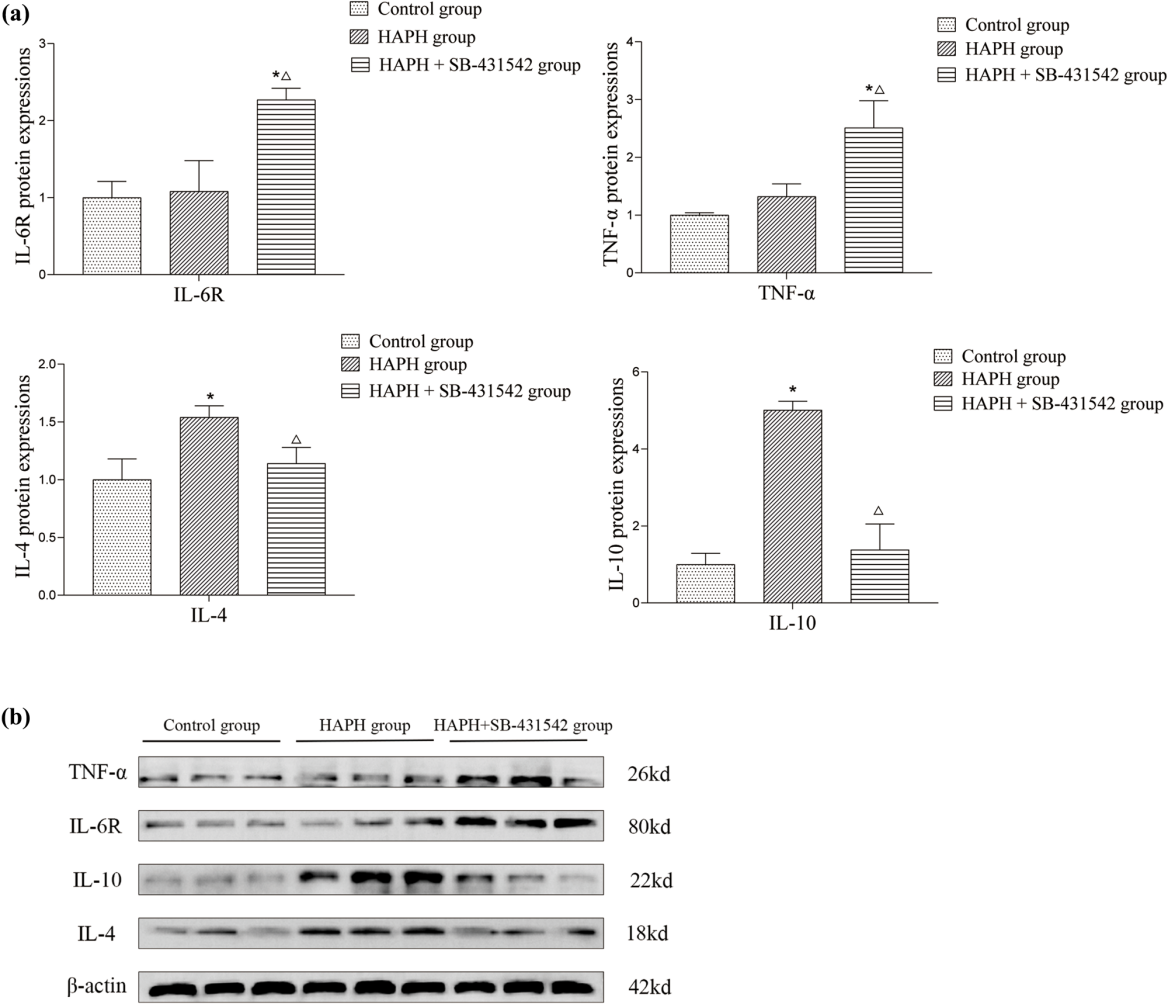
Quantitative analysis of IL-6R, TNF-α, IL-4, and IL-10 expression levels in lung tissue (a). Representative western blot bands of target proteins (b). Values are presented as mean ± standard deviation. *p < 0.05 compared to the control group; Δ*p* < 0.05 compared to the HAPH group. Abbreviations: IL-6R, interleukin 6 receptor; TNF-α, tumor necrosis factor alpha; IL-4, interleukin 4; IL-10, interleukin 10; SD, standard deviation; HAPH, high-altitude pulmonary hypertension.

Figure 5a and b displays the western blot analysis of pro-inflammatory markers IL-6R and TNF-α, alongside anti-inflammatory markers IL-4 and IL-10, in lung tissues. In the HAPH group, anti-inflammatory cytokines IL-4 and IL-10 were significantly elevated relative to the control group, whereas IL-6R and TNF-α levels remained unchanged. In the HAPH + SB-431542 group, IL-4 and IL-10 expression levels remained unchanged, while IL-6R and TNF-α levels increased. In the HAPH + SB-431542 group, IL-4 and IL-10 expression was significantly reduced, while IL-6R and TNF-α expression was significantly elevated compared to the HAPH group. The study demonstrated that high-altitude-like low-pressure and hypoxic conditions significantly elevated anti-inflammatory cytokines IL-4 and IL-10 in rats. However, the TGF-β inhibitor SB-431542 mitigated these effects and concurrently enhanced the expression of pro-inflammatory markers IL-6R and TNF-α.

## 4 Discussion

This study demonstrated a notable rise in M2 macrophages and increased secretion of anti-inflammatory cytokines IL-4 and IL-10 in a rat model of HAPH. TGF-β is essential for inducing macrophage polarization to the M2 phenotype, which facilitates HAPH progression. Inhibiting TGF-β reduced macrophage polarization to the M2 phenotype and decreased downstream anti-inflammatory cytokine levels, indicating that these cytokines from M2 macrophages likely contribute significantly to vascular remodeling in HAPH. Although SB-431542 significantly reversed vascular remodeling (Figure 2), the limited mPAP reduction (Figure 1b) indicates that neurohormonal activation and other factors may sustain hypertensive states even after structural correction in advanced HAPH.

The increase in M2 macrophages in PH requires further study. M2 macrophages play important roles in tissue repair, immune regulation, and parasitic infections by secreting anti-inflammatory cytokines, growth factors, and chemokines; however, an abnormal increase in macrophages of this phenotype can lead to excessive pathological tissue repair, thereby contributing to the onset of various diseases. For example, the proliferation of M2 macrophages can promote the development of idiopathic pulmonary fibrosis through mechanisms such as epithelial–mesenchymal transition, and targeted interventions that reduce M2 macrophage numbers may effectively prevent development of the disease [9,10]. In glioblastoma, M2 tumor-associated macrophages enhance tumor cell growth by releasing cytokines and chemokines such as IL-8 and macrophage inflammatory protein 3 alpha [11]. M2 macrophages also promote collagen expression in fibroblasts, contributing to iatrogenic laryngotracheal stenosis [12]. An unusual rise in M2 macrophages has also been noted in PH progression. In a lycorine-induced rat model of PH, increased accumulation of macrophages around pulmonary vessels can slow PH progression [2]. The current findings align with research on other conditions such as chronic thromboembolic pulmonary hypertension (CTEPH) [13] and idiopathic pulmonary arterial hypertension (IPAH) [14], and other types of PH induced by lycorine and hypoxia in rat models [15], where notable increases in M2-like macrophages (CD206+ Arg1+) have been observed in the absence of significant changes in M1-like macrophages (CD86+ iNOS+) in HAPH.

TGF-β, a cytokine mainly produced by immune cells, is crucial for tissue repair and healing. M2 macrophage levels are closely linked to TGF-β concentrations in lung tissue. As one of the primary immune cells that secrete TGF-β, M2 macrophages can participate in pathophysiological responses by secreting the protein in large quantities [9], further driving the polarization of macrophages toward the M2 phenotype and enhancing the anti-inflammatory response [16]. In PH, elevated TGF-β expression contributes to various facets of pulmonary vascular remodeling, such as endothelial-to-mesenchymal transition [17], proliferation and migration of pulmonary artery smooth muscle cells [18], and the secretion of specific growth factors and cytokines [19]. These results align with recent findings of studies that have investigated mechanisms associated with anaplastic thyroid cancer [20] and ovarian cancer [21], in which the TGF-β–Smad2/Smad3 pathway has been shown to drive M2 macrophage polarization. This study demonstrates that in HAPH, the TGF-β–Smad2/Smad3 pathway induces macrophage polarization to the M2 phenotype, enhancing HAPH progression by elevating anti-inflammatory cytokines IL-4, IL-10, and TGF-β. Inhibiting TGF-β expression with a specific inhibitor reduced HAPH development, underscoring its crucial role. Nonetheless, the complexity and diverse effects of the TGF-β signaling pathway *in vivo* require additional research to clarify the exact mechanisms involved.

In this study, we chose to use only male rats primarily to avoid the potential interference of estrogen on the experimental outcomes. Estrogen can influence immune responses, metabolic processes, and the TGF-β signaling pathway, which in turn affects the polarization of M2 macrophages and cytokine secretion. Therefore, to simplify the experimental design and minimize these variables, we used male rats. However, gender may differ in some physiological and pathological processes. Studies have shown that male and female rats may exhibit different characteristics in immune responses, cardiovascular diseases, and the progression of pulmonary arterial hypertension (HAPH) [22]. Gender differences may affect cytokine levels and immune cell functions, thereby influencing disease progression [23]. Therefore, future research should consider including female rat cohorts to assess the impact of gender on HAPH progression and immune cell changes.

In HAPH, elevated M2 macrophages contribute to pulmonary vascular remodeling by secreting anti-inflammatory cytokines like IL-4 and IL-10. These cytokines induce abnormal proliferation and apoptosis in smooth muscle cells, endothelial cells, and fibroblasts, while also increasing extracellular matrix secretion. IL-4 is an essential anti-inflammatory cytokine that significantly influences PH development. IL-4 in PH research reveals its perplexing diversity, and clinical studies that have investigated different types of PH have shown that while IL-4 levels are diminished in CTEPH and IPAH, they are enhanced in congenital heart disease-associated PH (CHD-PH) [24]. Furthermore, elevated IL-4 levels have been reported in asthma-induced PH in both clinical populations and in mouse models, as well as in schistosomiasis-induced PH in mice [25,26]. In the present study, elevated levels of IL-4 were also observed; however, administration of a TGF-β inhibitor led to a decrease in IL-4 expression, suggesting that TGF-β inhibits the differentiation of T helper (Th) to T helper 2 (Th2) cells, thereby reducing the downstream secretion of IL-4 [27]. Elevated IL-10 levels are observed across all PH types, such as IPAH, CHD-PH, connective tissue disease-associated PH, and CTEPH, aligning with this study’s findings. This suggests that increased IL-10 levels in HAPH significantly contribute to pulmonary vascular remodeling through their immunosuppressive and anti-inflammatory effects [24].

In contrast to the alterations in anti-inflammatory cytokines, no abnormal changes in the expression levels of the pro-inflammatory proteins IL-6R and TNF-α were observed in the HAPH group. A Mendelian randomization study based on aggregate data from multiple biobanks suggested that the IL-6 signaling pathway is not causally associated with the risk of PH [28]. A phase II non-blinded clinical trial on the IL-6R antagonist tocilizumab in PH patients found no impact on pulmonary vascular resistance at 6 months, despite reduced C-reactive protein and increased plasma IL-6 levels [29]. This is consistent with the lack of significant changes in IL-6R and TNF-α expression in HAPH rat lung tissues observed in this study. Surprisingly, administering the TGF-β inhibitor elevated IL-6R and TNF-α expression, but its role in alleviating HAPH is unclear. This result could be related to a disruption in the macrophage polarization equilibrium, alterations in cellular signaling pathways, and/or changes in regulatory feedback mechanisms, meriting further exploration and study.

Investigating macrophage polarization regulatory mechanisms and the interplay between TGF-β, other cytokines, and signaling pathways may offer new insights into HAPH pathogenesis. Future research could focus on a detailed examination of the dynamic changes in macrophage polarization, the creation of targeted therapies for the TGF-β signaling pathway, and the validation of these findings in larger clinical studies to identify more effective prevention and treatment strategies for HAPH.
